# The impact of affective and negative symptoms on the development of psychosis in a six-year follow-up of a community-based population

**DOI:** 10.1007/s00127-024-02785-0

**Published:** 2024-11-07

**Authors:** Ceylan Ergül, Tolga Binbay, Umut Kırlı, Hayriye Elbi, Köksal Alptekin, Jim van Os, Marjan Drukker

**Affiliations:** 1 Department of Psychiatry, Uskudar University, Istanbul, Turkey; 2 Department of Psychiatry and Neuropsychology, School for Mental Health and Neuroscience, Maastricht University Medical Center, Maastricht, The Netherlands; 3 Private Practice, İzmir, Turkey; 4 Institute On Drug Abuse, Toxicology and Pharmaceutical Science, Ege University, Izmir, Turkey; 5 Department of Psychiatry, Faculty of Medicine, Ege University, Izmir, Turkey; 6 Department of Psychiatry, Faculty of Medicine, Dokuz Eylul University, Izmir, Turkey; 7 Department of Psychiatry, UMC Utrecht Brain Centre, University Medical Centre Utrecht, Utrecht University, Utrecht, The Netherlands; 8 Department of Psychosis Studies, Institute of Psychiatry, Psychology & Neuroscience, King’s College London, London, UK

**Keywords:** Psychotic experiences, Psychotic disorders, Clinical high risk, Transition

## Abstract

**Purpose:**

The Clinical High Risk (CHR) concept has a limited transition risk to psychotic disorders (PD). This study investigates the association between affective and negative symptoms, currently not included in the CHR concept, and the risk of transition to PD in a community-based population of 2185 participants in Turkey.

**Methods:**

Participants were assessed twice over six years using a multistage sampling technique. Two separate linear regression analyses were conducted on data from both assessments, investigating the relationship between affective and negative symptoms, subclinical and clinical psychotic experiences (PE) and progression to PD.

**Results:**

The overall transition rate to PD was 1.3%. The analysis showed no increased risk of developing PD for the 'subclinical PE only' group at follow-up, compared to the 'no PE' group. However, being classified as having 'clinical PE only' (OR: 6.23; *p* = 0.010) and 'clinical PE + affective/negative symptoms' (OR: 8.48; *p* = 0.001) at baseline was associated with an increased risk of developing PD at follow-up. The presence of 'affective/negative symptoms' at baseline was associated with an increased risk of incident subclinical PE (RR: 1.98; *p* = 0.001), incident clinical PE (RR: 3.14; *p* = 0.001), and incident PD (RR: 4.21; *p* = 0.030) at follow-up.

**Conclusion:**

The results confirm the significance of the baseline severity of positive symptoms in predicting the transition to PD and suggest that both positive and affective/negative symptoms impact the transition risk to PD and incident psychotic symptoms. This highlights the potential utility of defining CHR groups based on a combination of positive, affective, and negative symptoms.

**Supplementary Information:**

The online version contains supplementary material available at 10.1007/s00127-024-02785-0.

## Introduction

The concept of Clinical High Risk (CHR) has been extensively studied in the field of psychosis research for the past 30 years, with the prospect of identifying individuals at risk of developing a psychotic disorder (PD) [1]. The traditional definition of the CHR group is primarily based on the presence and severity of positive symptoms, help-seeking behaviour, and impairment in the level of functioning [2]. However, recent studies show that the two-year rate of variably defined ‘transition’ to psychosis in the CHR group remains at around 20% [3]. Recent research has suggested that other symptoms, such as negative and affective symptoms, may also contribute to the risk of developing psychosis [4–6]. Therefore, a more comprehensive assessment of symptoms may be needed to improve the accuracy of identifying individuals at risk of developing psychosis.

CHR continues to be a prominent focus in clinical psychosis research aiming to define individuals at risk of developing a PD. Existing research has largely focused on positive symptoms while neglecting the interactions with other symptom dimensions that are often present in PD, particularly negative and affective symptoms. However, positive symptoms are not always the first indicators of the development of PD. The prodrome of PD is characterized by depressive or negative symptoms and functional decline [7, 8]. Furthermore, there is considerable overlap between psychotic and affective disorders, despite the two being categorized as separate constructs [9, 10]. Similarly, non-psychotic symptoms are prevalent in CHR groups [11]. In addition to this, recent studies have questioned the validity of the CHR paradigm due to relatively low rates of transition to PD [12, 13]. Although previous research primarily focused on homotypical positive symptoms and the CHR criteria as predictors of transition to psychosis, recent studies have highlighted the importance of heterotypical constructs in predicting the risk of developing a PD [14]. Decline in global functioning and negative symptoms, especially social anhedonia and withdrawal, were identified as important predictors of transition to psychosis [15, 16]. Similarly, Hasmi and colleagues used a combined phenotype of psychotic experiences (PE) and non-psychotic disorders to identify individuals at risk of developing a PD, arguing that non-psychotic disorders should be considered an integral component of the risk for transition to a PD rather than a comorbidity to be excluded as per previous studies [5]. These findings suggest that the integration of affective and negative symptoms into the CHR definition may be required to increase the precision – i.e. increasing specificity albeit at the expense of sensitivity – of identifying people who will transition to PD.

The present paper is part of The Izmir Mental Health Survey for Gene-Environment Interaction in Psychoses (TürkSch). TürkSch screened for subclinical PEs, which are psychotic symptoms not leading to any distress, impairment or help-seeking; clinical PEs, meaning a psychotic symptom causing distress, impairment or help-seeking but not meeting the criteria of a PD; and PDs in residents of various neighbourhoods within an urban environment [17]. The baseline data revealed that almost 25% of the general population had experiences meeting the criteria for extended psychosis phenotype [18]. Previous analyses of the six-year follow-up data showed that the six-year rate of PD was 7%, whereas the six-year rate of affective disorders without psychotic features was around 43% for participants who had clinical PE at baseline [19]. Also, it was reported that PE and mood episodes bidirectionally predicted each other [20]. Aiming to build on previous findings of the TürkSch, the current paper investigates the association between affective and negative symptoms and the risk of clinical transition to psychosis as well as incident psychosis, in a six-year follow-up of a community-based population.

## Methods

### Study design and data collection

This research is a part of the TürkSch project, which was supported by the 1001 program of the Scientific and Technological Council of Turkey (Project numbers: 107S053 and 112S476). The Ege University ethics committee gave its approval, and all participants provided written informed consent.

TürkSch is a longitudinal, prospective study which aims to screen and follow up several groups of mental symptoms as well as individual, family and neighbourhood-level variables [17]. TürkSch consisted of two assessments, approximately six years apart (mean follow-up time: 6.11 ± 0.94 years): baseline (T1: 2007–2009) and follow-up (T2: 2013–2015). Each assessment involved a number of stages for collecting data (T1: Stages 1–3; T2: Stages 4–5). The data collected at stages 1 and 4 were used in the current paper.

The data was collected from the larger İzmir metropolitan area, one of the most urbanised regions in Turkey. The baseline sample was selected using a multistage sampling procedure, stratified by urbanicity covering 11 districts and 302 neighbourhoods. The households were visited in person by trained lay interviewers. The Kish within-household sampling method [21] was used to randomly choose a household member between 15 and 64 years old. Individuals diagnosed with a psychotic disorder were exempted from the randomization procedures and directly recruited for the study [17]. Originally designed as a cross-sectional study, the first stage included an evaluation of positive, negative, and affective symptoms as well as sociodemographic data at the individual level (T1, Stage 1, N = 4011). Addresses of all T1 participants were revisited in person six years after baseline. All stage 1 participants who could be reached six years later (T2, Stage 4, N = 2185) were surveyed to collect data on psychopathology as well as changes in sociodemographic features. Details of all stages have been described in previously published papers [17, 19].

A total of 1826 participants (45.6%) out of the initial 4011 interviewed at baseline could not be reached at follow-up. A separate investigation of the associations with non-contact and refusal in the longitudinal follow-up has been analyzed and published elsewhere [22]. The analysis revealed that older and lower-educated individuals had a lower probability of non-contact. Additionally, individuals with mental health conditions exhibited lower refusal rates [22].

### Assessment of the clinical variables

Positive (hallucinations, delusions – as well as associated impairment), negative and affective symptoms were assessed using the relevant sections of the Turkish version of the Composite International Diagnostic Interview (CIDI) 2.1 [23]. At T1, the assessed time period was lifetime; at T2, it was the last six years. The CIDI is a structured interview designed to be used by both clinicians and trained interviewers [24]. It was developed by the World Health Organisation to be used in epidemiological studies of mental disorders [25], and it helps diagnose various mental disorders in accordance with the criteria of the ICD-10 [26] and DSM-IV [27]. Almost all sections of the CIDI have excellent inter-rater reliability, with κ values ranging between 0.67 and 0.97 [28].

### Assessment of the positive symptoms

Psychotic symptoms were rated using the 19 symptoms in the CIDI section G. Every time a participant mentioned a CIDI psychotic symptom, the interviewer asked them for an example, which was then written down verbatim to be discussed later with a psychiatrist. The interviewer then noted down how likely it was that this particular experience was not psychotic but plausibly related to an actual incident that had happened. The psychiatrist examined all CIDI interviews and re-contacted any individual who had endorsed a psychotic symptom but may have actually been reporting a plausible non-psychotic event. Almost 6% of the sample was contacted by telephone for this reason.

The presence of a psychotic symptom which did not lead to any distress, impairment or help-seeking was named ‘subclinical PE’. If the participant endorsed one or more CIDI psychosis items in addition to the endorsement of any of the seven CIDI impairment items, indicating a high level of frequency, duration, help-seeking, severity, or psychosocial impairment associated with these PE, a second diagnostic assessment was conducted for those who had never been diagnosed with a psychotic illness. The study team contacted each of these people again and encouraged them to participate in a clinical assessment using the Structured Clinical Interview (SCID) for DSM-IV (Spitzer et al. 1992). A clinical psychologist or psychiatrist conducted a SCID interview at the patient's home or in the hospital before making the final diagnosis. Several case identification steps were used to identify participants who meet the criteria for PD. The first phase was asking the person and their family whether the participant had ever received treatment for a mental health issue and/or been given a diagnosis of a psychiatric disorder. If this were the case, the participant was asked for permission to get in touch with the medical expert who had been involved in the treatment in order to confirm the diagnosis. If the participant did not meet the criteria of a PD, but had a psychotic symptom causing distress, impairment or help-seeking, then this was named ‘clinical PE’.

### Assessment of the affective and negative symptoms

Participants who rated positive on any one of the depression or mania items were included in the affective symptoms group. Depressive symptoms were assessed using the 10 symptoms in the CIDI section E. Manic and hypomanic symptoms were assessed using the 12 symptoms in the CIDI section F. Four items of the CIDI section P, a section on interviewer observations, were used to assess flat affect, slow speech, poverty of speech, and impaired ability to initiate activity. Participants who rated positive on any one of those items were included in the negative symptoms group. Participants in the affective and negative symptoms groups were combined to form the 'affective/negative symptoms' group.

Participants in the affective and negative symptom groups were combined to form the 'affective/negative symptoms' group for several reasons. First, some symptoms (anhedonia, amotivation, and decrease in attention) form part of both depressive and negative symptomatology. Second, the power of the analyses would be low if performed separately for affective and negative symptoms due to the low number of participants. Finally, the rate of overlap between participants who had affective and negative symptoms was high as quantified below.

### Assessment of other clinical features

A sociodemographic survey was used at both T1 and T2. Ethnicity was dichotomised into Turkish and non-Turkish; cannabis use into users and non-users; childhood adversity and trauma into none versus at least once. Childhood adverse life events were separation from parents for at least three months between the ages of 0–15 years, divorce of parents, and the death of any parent. Traumatic events were 'being physically attacked or assaulted', sexual molestation, rape, life-threatening accident, fire, natural disasters, witnessing someone being badly injured or killed and war experience. Family history of mental disorder was recoded into four categories (none, other/unknown, common mental disorder, severe mental disorder). Family history of common mental disorder included any diagnosis of depression, anxiety, conversion or somatisation in the absence of severe mental disorder among the parents or siblings of the participant. Family history of severe mental disorder was coded positive if any of the participant's first-degree relatives had been diagnosed with PD or bipolar disorder, had died because of suicide or had been admitted to a psychiatric inpatient unit.

### Statistical analyses

All analyses were performed using Stata, version 13.1 (StataCorp, 2013). Data from 2185 participants who were interviewed at both T1 and T2 were included in the analyses.

**Fig. 1:**
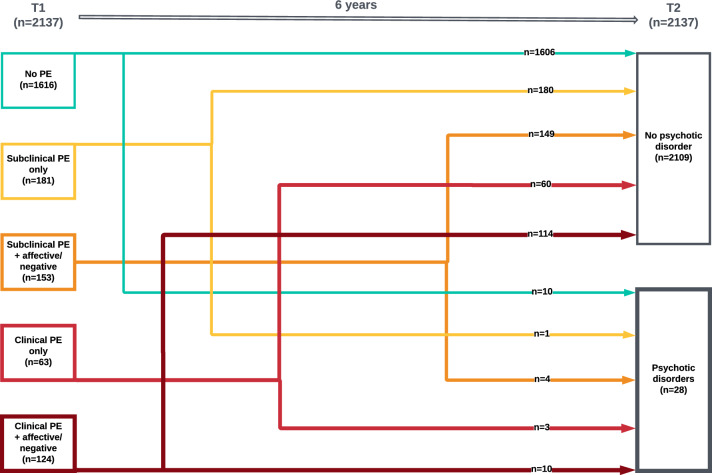
Dynamic transitions over time within the extended psychosis spectrum and groups defined for analysis (*PE:* psychotic experiences)

In the first analysis, we tested for association with transition to PD using logistic regression. Excluded were 48 participants with PD at baseline. The dependent variable was having a PD at follow-up. The independent variable was baseline symptomatology recoded into 5 categories: no PE (reference category), subclinical PE, subclinical PE + affective/negative symptoms, clinical PE and clinical PE + affective/negative symptoms (Fig. 1).

**Fig. 2:**
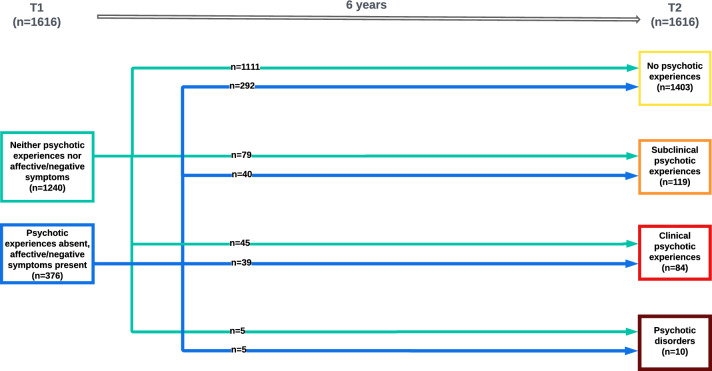
Dynamic transitions over time of baseline non-psychotic symptomatology into new psychotic experiences or psychotic disorders

In the second analysis, we tested the association between affective/negative symptoms and incident PE and PD. We used a variant of logistic regression analysis, multinomial logistic regression, allowing a categorical dependent variable (the mlogit routine in Stata, version 13.1). We excluded 569 participants who had a PE or PD at baseline. The dependent variable included 4 levels of incident PE defined using the follow-up assessment: no PE, subclinical PE, clinical PE, and PD. The independent variable was baseline symptomatology recoded into 2 categories: neither PE nor affective/negative symptoms (reference category), no PE but affective/negative symptoms (Fig. 2).

Gender, age, ethnicity, cannabis use, childhood adversity, trauma and history of mental illness in the family were added to all regression models as covariates. In the second analysis, family history of mental disorder had to be dichotomised into two (none versus present) due to the low number of cases in subgroups. In addition to the main analyses, we also performed sensitivity analyses using affective and negative symptom groups separately.

## Results

### Baseline characteristics

At baseline, the mean age of the participants was 38.6 ± 13.3 (15–65). The majority of the 2175 participants were female (59.4%), married (70.4%), of Turkish ethnicity (72.0%), and educated for ≤ 8 years (60.5%). The proportion of participants who used cannabis was 2.9%. The rate of childhood adversity was 14.4%, and the rate of traumatic events was 36.7%. A family history of common mental disorder was seen in 11.5%, whereas a family history of severe mental disorder was seen in 2.9% of the participants. 5.6% had other/unknown mental disorders in their families.

### Positive, affective and negative symptoms at baseline

**Fig. 3:**
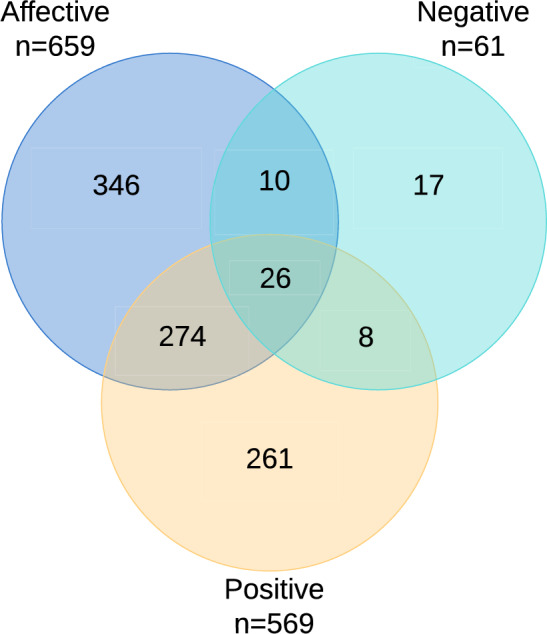
Overlap between positive, affective and negative symptom groups

The distribution of positive symptoms in the 2185 participants was: 1616 (73.9%) had no positive symptoms, 334 (15.3%) had subclinical PE, 187 (8.6%) had clinical PE, and 48 (2.2%) had PD. The number of participants with affective symptoms was 659 (30.2%), whereas the number of those with negative symptoms was 61 (2.8%). The overlap between the three symptom groups is presented in Fig. 3. Thirty-six participants had both negative and affective symptoms, meaning that 59.0% with negative symptoms also had affective symptoms. We also checked for overlap between negative and positive symptoms. 34 (55.7%) of participants with negative symptoms also had positive symptoms. Regarding the severity of affective symptomatology, 182 (27.6%) of participants with affective symptoms met the criteria for major depressive disorder.

### Transition to PD

The six-year overall rate of transition to PD was 1.3%. The transition rate from baseline 'no PE' to PD was 0.6%, whereas it was 3.5% for all participants with baseline PE. In various PE groups, the transition rates to PD ranged from 0.6% (subclinical PE only) to 2.6% (subclinical PE plus affective/negative symptoms) to 4.8% (clinical PE only) and to 8.1% (clinical PE plus affective/negative symptoms).

The results of the initial analysis revealed that when comparing membership in various 'PE' (psychotic experiences) groups to being in the 'no PE' group, a dose–response relationship was evident. The Odds Ratios (ORs) ranged from 0.87 (p = 0.898; subclinical PE only) to 3.22 (p = 0.057; subclinical PE plus affective/negative symptoms) to 6.23 (p = 0.010; clinical PE only) and finally to 8.48 (p = 0.001; clinical PE plus affective/negative symptoms), as shown in Table 1. Cannabis use and family history of common mental disorders were also independently associated with an increased risk of transition to PD (Table 1).

**Table 1:** Results of the logistic regression analysis of the association between baseline positive, affective and negative symptomatology and clinical characteristics with transition to PD at follow-up

	Transition to PD			
	OR	95% CI	z	*p*
**Categories at T1**				
No PE	Ref			
Subclinical PE only	0.87	0.11–6.94	– 0.13	0.898
Subclinical PE + aff/neg	3.22	0.96–10.76	1.90	0.057
Clinical PE only	6.23	1.54–25.18	2.57	**0.010**
Clinical PE + aff/neg	8.48	3.10–23.17	4.17	**0.001**
**Gender**				
Male	Ref	–		
Female	0.92	0.39–2.16	– 0.20	0.840
**Age**				
15–30	Ref	–		
31–45	0.60	0.24–1.49	– 1.10	0.271
46–65	0.37	0.12–1.18	– 1.67	0.095
**Ethnicity**				
Turkish ethnicity	Ref			
Non-Turkish ethnicity	0.82	0.34–1.99	– 0.44	0.663
**Cannabis use**	5.70	1.95–16.68	3.18	**0.001**
**Adversity**	1.53	0.58–4.03	0.86	0.392
**Trauma**	0.92	0.40–2.12	– 0.19	0.846
**Family history of mental disorder**				
None	Ref			
Unknown/other disorder	1.83	0.38–8.75	0.75	0.453
Common mental disorder	3.44	1.40–8.40	2.71	**0.007**
Severe mental disorder	2.59	0.49–13.71	1.12	0.262

Significant *p* values are in bold (*p* < 0.05)

*PE* Psychotic Experiences, *PD* Psychotic Disorders, *OR* Odds Ratio, *CI* Confidence Interval

### Incident PE/PD

Results of the second analysis (Table 2) showed that being in the ‘affective/negative symptoms’ group at baseline was associated with an increased risk of incident subclinical PE (RR: 1.96; *p* = 0.001), incident clinical PE (RR: 3.14; *p* = 0.001) and incident PD (RR: 4.21; *p* = 0.030) at follow-up compared with being in the ‘neither PE nor affective/negative symptoms’ group. Cannabis use and younger age were also independently associated with an increased risk of incident subclinical PE (Table 2).

**Table 2:** Results of the multinomial logistic regression analysis of the association between baseline affective/negative symptoms and clinical characteristics with incident PE and PD at follow-up

	Incident Subclinical PE				Incident Clinical PE				Incident PD			
	RR	95% CI	z	*p*	RR	95% CI	z	*p*	RR	95% CI	z	*p*
Categories at T1												
No PE nor aff/neg symptoms	Ref				Ref				Ref			
Affective or negative symptoms	1.98	1.30–3.00	3.20	**0.001**	3.14	1.98–5.00	4.84	**0.001**	4.21	1.15–15.36	2.17	**0.030**
Gender												
Male	Ref	–			Ref				Ref			
Female	0.86	0.58–1.28	– 0.72	0.496	1.23	0.75–2.00	0.82	0.412	0.44	0.12–1.67	– 1.20	0.229
Age												
15–30	Ref	–			Ref				Ref			
31–45	0.59	0.37–0.93	– 2.29	**0.022**	0.84	0.49–1.44	– 0.63	0.528	0.28	0.05–1.46	– 1.51	0.131
46–65	0.51	0.32–0.82	– 2.81	**0.005**	0.58	0.33–1.04	– 1.81	0.070	0.28	0.05–1.44	– 1.53	0.127
Ethnicity												
Turkish ethnicity	Ref				Ref				Ref			
Non-Turkish ethnicity	0.77	0.49–1.21	– 1.13	0.257	0.78	0.45–1.32	– 0.94	0.347	0.76	0.16–3.68	– 0.34	0.734
Cannabis use	3.19	1.17–8.70	2.27	**0.023**	2.63	0.71–9.80	1.44	0.149	3.58	0.37–34.86	1.10	0.272
Adversity	1.46	0.87–2.45	1.42	0.155	1.53	0.84–2.78	1.39	0.164	0.89	0.11–7.29	– 0.11	0.912
Trauma	1.32	0.88–1.96	1.35	0.177	1.29	0.81–2.06	1.06	0.291	0.76	0.19–3.10	– 0.38	0.705
Family history of mental disorder												
None	Ref				Ref				Ref			
Present	0.82	0.49–1.37	– 0.76	0.445	0.93	0.53–1.64	– 0.25	0.800	1.72	0.42–7.05	0.75	0.454

Significant *p* values are in bold (*p* < 0.05)

*PE* Psychotic Experiences, *PD* Psychotic Disorders, *RR* Relative Ratio, *CI* Confidence Interval

The results of the sensitivity analyses which were performed using affective and negative symptom groups separately are reported in Supplement Tables 1–4.

## Discussion

The present study aimed to investigate the role of affective and negative symptoms in the development of psychosis in a community-based population over a six-year period. The main finding was that the severity of positive symptoms at baseline was significantly associated with the transition to psychosis and that the co-presence of affective and negative symptoms strengthened this association. In addition to this, the presence of affective and negative symptoms at baseline was associated with incident psychosis. These results, therefore, suggest that defining CHR groups based on a combination of positive, affective, and negative symptoms instead of solely focusing on positive symptoms can be useful in creating improved prediction.

### Transition to PD

The first analysis revealed that having ‘clinical PE only’ and also ‘clinical PE + affective/negative symptoms’ at baseline was associated with an increased risk of having a PD at follow-up compared with the ‘no PE’ group. Additionally, there was a suggestive association with the ‘subclinical PE + affective/negative symptoms’ group. The nominal non-significance of this association may be due to the low number of participants who transit to PD during the six-year follow-up. It is noteworthy to mention that these associations were in the same direction, and the relative risk of transition to PD gradually increased parallel to the order of the baseline categories we formed. This gradual increase might imply that this order is clinically relevant for the transition to PD.

### Incident PE/PD

The second analysis revealed that having affective and negative symptoms at baseline increased the risk of incident subclinical PE, clinical PE and PD at follow-up compared with being in the ‘neither PE nor affective/negative symptoms’ group. The relative risk ratio gradually increased parallel to the order of the follow-up categories we formed. This might emphasize the importance of affective and negative symptoms in the development of clinically more severe psychotic states. The potential mechanism leading to this finding might be that the prodromal period before psychosis may begin with negative and affective symptoms without the presence of PEs. This may also imply that the categorical definitions of psychiatric disorders are imprecise as the symptom mix may be time-dependent.

### Comparison with previous studies

Our findings are in line with previous research that has identified positive symptoms as a key homotypical risk factor for the development of psychosis [2, 29]. In addition to this, our results imply that heterotypical affective and negative symptoms make an important contribution to predicting transition when combined with positive symptoms. This finding is consistent with the results of some previous CHR studies. The 3-year follow-up of 285 patients from the Dutch Prediction of Psychosis Study revealed that the transition group had more social anhedonia and withdrawal at baseline [15]. A review of the studies about the transition to PD published in 2015 and 2016 found that the risk of transition was higher in participants who had anhedonia and lower social functioning [30]. A meta-analysis by Oliver et al. reported that negative symptoms are associated with the risk of transition to psychosis [16]. In a 24-month follow-up study of 71 participants at CHR for psychotic disorders, the participants were assessed according to the Hierarchical Taxonomy of Psychopathology (HiTOP). The authors found that converters were elevated at baseline on the negative symptom dimension compared with the others [31]. In a 24-month follow-up of 331 UHR subjects, a past depressive disorder was found to be a risk factor for an increasing trajectory of attenuated psychotic symptoms [32].

We could not find any other study to directly compare our finding that baseline affective and negative symptoms in the absence of positive symptoms are associated with incident psychosis. However, our results support a study by Guloksuz and colleagues showing that preceding diagnoses of mood disorders were associated with an increased risk of incident clinical psychosis [6]. Hasmi and colleagues similarly emphasized the importance of non-psychotic disorders in transition and outcome, comparing the incidence of PE with and without non-psychotic disorders [5]. It is important to note that our study differs in its methodology, as it utilizes affective and negative symptoms rather than categorically defined disorders. This approach allows for the identification of the significance of less severe symptomatology. Furthermore, future studies with a larger participant pool may benefit from stratifying according to the severity of negative and affective symptoms.

### Demographic and background characteristics

In terms of demographic and background characteristics, our findings are generally in line with the literature [33, 34]. However, it is important to mention that we did not find any associations with gender, whilst male gender was more prevalent in a meta-analysis of CHR studies [35]. The difference might be due to the fact that this is community-based research, whereas the majority of other studies are based on clinical samples in the context of help-seeking. Also, the mean age is higher compared to other CHR studies, possibly leading to a lower rate of incident PD and also transition to PD because lower cohort age is found to be correlated with a higher prevalence of psychosis [33]. Another important point is that a family history of common mental disorder was significantly associated with the transition to PD, whereas a family history of severe mental disorder was not. This might be due to the low number of participants who had a family history of severe mental disorder. On the other hand, it might also be because we used baseline symptoms and not baseline disorders in our analyses. This finding might imply that similar to the importance of affective and negative symptoms in addition to the positive symptoms, a family history of common mental disorder might also be more important than is traditionally considered. Other studies have shown that baseline cognitive functioning [36] and global functional impairment [16] can predict transition to psychosis, however, we could not control for these factors because the dataset does not include data on cognition or functioning.

### Methodological issues

TürkSch was carried out in a highly urbanized area of Turkey, using a large community-based sample that was representative of the general population. Our study has several strengths, including the collection of high-quality data from a large number of participants at two different time points through house visits conducted by trained interviewers. Our longitudinal design enabled us to create categories based on dynamic transitions within the extended psychosis spectrum, which is more useful than relying solely on categories based on cross-sectional data. Furthermore, using a general population sample allowed us to capture individuals who may not be included in a clinical sample, and the random collection of data across a geographical area minimized help-seeking bias and revealed the spectrum, including subclinical manifestations.

It is important to consider some limitations of our study. First, the data were collected by visiting randomly chosen households. Therefore, homeless and institutionalised people who are more likely to have PD were not included. Second, the households were visited during the day, thus interviewers found more women than men at home. As a result, especially young working men were underrepresented in the sample. Third, psychotic patients may be unaware of their symptoms or may underreport them. Clinical interviews were performed and health records were screened for additional information to minimise this bias. Fourth, the number of participants who had PD at T2 was relatively low despite a risk enrichment strategy of not using a randomization method but directly recruiting an individual if they had a diagnosis of PD. The low number of transition to psychosis may have decreased the power of some of our analyses.

Almost half of the participants at baseline could not be reached at follow-up. The attrition analysis revealed that older and lower-educated individuals had a lower probability of non-contact [22]. The reason for this might be that the data collection was performed during working hours by house visits. Younger and higher-educated people were possibly at work, indicating a certain level of functioning. Additionally, participants with a mental health problem had lower refusal rates contrary to expectations [22]. Both findings imply that those who could not be reached at follow-up were healthier. Therefore, we can interpret that the employed sampling method was able to identify participants with symptoms.

In addition to these methodological issues, another important point is that we combined affective and negative symptoms into one group for the analyses. There were several reasons for this. First, the rate of negative symptoms was very low, therefore analysing them separately would decrease the power of the analyses. Second, negative symptoms overlapped with affective symptoms to a high degree (Fig. 3). Therefore, it would not be possible to exclude the effect of affective symptoms even if the negative symptoms were analyzed separately. Third, it is clinically not easy to correctly differentiate between negative and depressive symptoms. This is supported by findings in a study of CHR participants [31]. The depression dimension partially loaded onto the negative symptom dimension and the hypomania dimension partially negatively loaded onto the negative symptom dimension [31]. For these reasons, we chose to combine affective and negative symptoms into one group. The findings of the sensitivity analyses using only the affective symptom group are similar to those of the main analyses. The results of the analyses using only the negative symptom group are incomplete due to the low number of data in some categories Therefore, the results can not be interpreted. It is further worth noting that some negative symptoms might be associated with cognitive deficits [37], and a decline in cognitive functioning is seen in CHR states [38] or prodromal psychotic episodes [39]. However, it is important to acknowledge that the TürkSch does not include data about cognitive functioning. Separate evaluation of affective, negative and cognitive symptoms in a larger dataset will yield a more nuanced understanding of the subject in future studies.

### Clinical implications

Currently, individuals at CHR for psychosis can be identified by standardized psychometric instruments such as the Comprehensive Assessment of At-Risk Mental States (CAARMS) [40] and the Structured Interview for Psychosis-Risk Syndromes (SIPS) [41]. Although the SIPS includes evaluation of the negative and general symptoms, the risk stratification relies on positive symptoms, level of functioning and family history. Our finding of a dose–response relationship for transition to PD might imply that affective and negative symptoms should also be taken into consideration for risk stratification. The presence of cooccurring affective and negative symptoms may be used as an indicator in addition to the current classifications.

## Conclusion

In conclusion, our study highlights the importance of considering positive, affective and negative symptoms altogether in the identification of individuals at risk for developing psychosis. This approach may improve the accuracy of risk prediction and facilitate early intervention efforts aimed at preventing the onset of PD.

## Supplementary Information

Below is the link to the electronic supplementary material.

Supplementary file1 (DOCX 22 KB)

Supplementary file2 (DOCX 27 KB)

Supplementary file3 (DOCX 22 KB)

Supplementary file4 (DOCX 27 KB)

## Data Availability

No datasets were generated or analysed during the current study.
