# Supplementary material for: The impact of affective and negative symptoms on the development of psychosis in a six-year follow-up of a community-based population
Source: Soc Psychiatry Psychiatr Epidemiol. 2024 Nov 7;60(6):1357–66. doi: 10.1007/s00127-024-02785-0 (PMC12162375; doi:10.1007/s00127-024-02785-0)
Supplement: Supplementary file 1 — Supplementary file1 (DOCX 22 KB) [file 127_2024_2785_MOESM1_ESM.docx]

**Supplement Table 1:** Results of the logistic regression analysis of the association between baseline positive and affective symptomatology and clinical characteristics with transition to PD at follow-up

|  | **Transition to PD** | | | |
| --- | --- | --- | --- | --- |
|  | **OR** | 95% CI | **z** | *p* |
| **Categories at T1** |  |  |  |  |
| No PE | ref |  |  |  |
| Subclinical PE only | **0.87** | 0.11-6.94 | **-0.13** | 0.898 |
| Subclinical PE+aff | **3.22** | 0.96-10.7 | **1.90** | 0.057 |
| Clinical PE only | **6.17** | 1.53-24.90 | **2.56** | **0.011** |
| Clinical PE+aff | **8.53** | 3.11-23.35 | **4.17** | **0.001** |
| **Gender** |  |  |  |  |
| Male | ref | - |  |  |
| Female | **0.91** | 0.39-2.15 | **-0.21** | 0.837 |
| **Age** |  |  |  |  |
| 15-30 | ref | - |  |  |
| 31-45 | **0.60** | 0.24-1.50 | **-1.09** | 0.274 |
| 46-65 | **0.37** | 0.12-1.19 | **-1.67** | 0.095 |
| **Ethnicity** |  |  |  |  |
| Turkish ethnicity | ref |  |  |  |
| Non-Turkish ethnicity | **0.82** | 0.34-2.00 | **-0.44** | 0.663 |
| **Cannabis use** | **5.69** | 1.94-16.66 | **3.17** | **0.002** |
| **Adversity** | **1.53** | 0.58-4.03 | **0.86** | 0.391 |
| **Trauma** | **0.92** | 0.40-2.11 | **-0.20** | 0.844 |
| **Family history of mental disorder** |  |  |  |  |
| None | ref |  |  |  |
| Unknown/other disorder | **1.82** | 0.38-8.74 | **0.75** | 0.455 |
| Common mental disorder | **3.44** | 1.40-8.40 | **2.71** | **0.007** |
| Severe mental disorder | **2.60** | 0.49-13.72 | **1.12** | 0.262 |

**PE**: Psychotic Experiences; **PD**: Psychotic Disorders; **OR**: Odds Ratio; **CI**: Confidence Interval
