# Supplementary material for: The impact of affective and negative symptoms on the development of psychosis in a six-year follow-up of a community-based population
Source: Soc Psychiatry Psychiatr Epidemiol. 2024 Nov 7;60(6):1357–66. doi: 10.1007/s00127-024-02785-0 (PMC12162375; doi:10.1007/s00127-024-02785-0)
Supplement: Supplementary file 2 — Supplementary file2 (DOCX 27 KB) [file 127_2024_2785_MOESM2_ESM.docx]

**Supplement Table 2:** Results of the multinomial logistic regression analysis of the association between baseline affective symptoms and clinical characteristics with incident PE and PD at follow-up

|  | **Incident Subclinical PE** | | | | **Incident Clinical PE** | | | | **Incident PD** | | | |
| --- | --- | --- | --- | --- | --- | --- | --- | --- | --- | --- | --- | --- |
|  | **RR** | 95% CI | **z** | *p* | **RR** | 95% CI | **z** | *p* | **RR** | 95% CI | **z** | *p* |
| **Categories at T1** |  |  |  |  |  |  |  |  |  |  |  |  |
| No PE nor affective symptoms | **ref** |  |  |  | **ref** |  |  |  | **ref** |  |  |  |
| Affective symptoms | **2.09** | 1.38-3.19 | **3.45** | **0.001** | **2.42** | 1.50-3.88 | **3.65** | **0.001** | **4.54** | 1.23-16.72 | **2.28** | **0.023** |
| **Gender** |  |  |  |  |  |  |  |  |  |  |  |  |
| Male | **ref** | - |  |  | **ref** |  |  |  | **ref** |  |  |  |
| Female | **0.85** | 0.57-1.27 | **-0.80** | 0.426 | **1.27** | 0.78-2.06 | **0.96** | 0.335 | **0.43** | 0.12-1.63 | **-1.25** | 0.213 |
| **Age** |  |  |  |  |  |  |  |  |  |  |  |  |
| 15-30 | **ref** | - |  |  | **ref** |  |  |  | **ref** |  |  |  |
| 31-45 | **0.59** | 0.37-0.92 | **-2.29** | **0.022** | **0.86** | 0.50-1.46 | **-0.57** | 0.570 | **0.28** | 0.05-1.46 | **-1.51** | 0.130 |
| 46-65 | **0.51** | 0.32-0.82 | **-2.78** | **0.005** | **0.60** | 0.34-1.07 | **-1.73** | 0.083 | **0.28** | 0.06-1.46 | **-1.51** | 0.131 |
| **Ethnicity** |  |  |  |  |  |  |  |  |  |  |  |  |
| Turkish ethnicity | **ref** |  |  |  | **ref** |  |  |  | **ref** |  |  |  |
| Non-Turkish ethnicity | **0.77** | 0.49-1.21 | **-1.14** | 0.252 | **0.77** | 0.45-1.30 | **-0.98** | 0.325 | **0.75** | 0.15-3.62 | **-0.36** | 0.717 |
| **Cannabis use** | **3.23** | 1.19-8.80 | **2.30** | **0.022** | **2.82** | 0.76-10.41 | **1.55** | 0.120 | **3.78** | 0.39-36.73 | **1.15** | 0.252 |
| **Adversity** | **1.47** | 0.88-2.47 | **1.45** | 0.198 | **1.52** | 0.84-2.76 | **1.39** | 0.164 | **0.90** | 0.11-7.35 | **-0.10** | 0.919 |
| **Trauma** | **1.30** | 0.87-1.94 | **1.29** | 0.198 | **1.32** | 0.83-2.11 | **1.17** | 0.240 | **0.74** | 0.18-3.01 | **-0.42** | 0.671 |
| **Family history of mental disorder** |  |  |  |  |  |  |  |  |  |  |  |  |
| None | **ref** |  |  |  | **ref** |  |  |  | **ref** |  |  |  |
| Present | **0.81** | 0.48-1.35 | **-0.81** | 0.417 | **0.96** | 0.55-1.70 | **-0.13** | 0.896 | **1.65** | 0.40-6.84 | **0.69** | 0.489 |

**PE**: Psychotic Experiences; **PD**: Psychotic Disorders; **RR**: Relative Ratio; **CI**: Confidence Interval
