# Supplementary material for: The impact of affective and negative symptoms on the development of psychosis in a six-year follow-up of a community-based population
Source: Soc Psychiatry Psychiatr Epidemiol. 2024 Nov 7;60(6):1357–66. doi: 10.1007/s00127-024-02785-0 (PMC12162375; doi:10.1007/s00127-024-02785-0)
Supplement: Supplementary file 3 — Supplementary file3 (DOCX 22 KB) [file 127_2024_2785_MOESM3_ESM.docx]

**Supplement Table 3:** Results of the logistic regression analysis of the association between baseline positive and negative symptomatology and clinical characteristics with transition to PD at follow-up

|  | **Transition to PD** | | | |
| --- | --- | --- | --- | --- |
|  | **OR** | 95% CI | **z** | *p* |
| **Categories at T1** |  |  |  |  |
| No PE | ref |  |  |  |
| Subclinical PE only | **2.10** | 0.70-6.94 | **1.32** | 0.186 |
| Subclinical PE+neg | * | * | ***** | * |
| Clinical PE only | **6.75** | 2.56-17.82 | **3.86** | **0.001** |
| Clinical PE+neg | **15.70** | 3.18-77.44 | **3.38** | **0.001** |
| **Gender** |  |  |  |  |
| Male | ref | - |  |  |
| Female | **0.96** | 0.41-2.26 | **-0.10** | 0.921 |
| **Age** |  |  |  |  |
| 15-30 | ref | - |  |  |
| 31-45 | **0.58** | 0.23-1.43 | **-1.18** | 0.237 |
| 46-65 | **0.36** | 0.11-1.13 | **-1.76** | 0.078 |
| **Ethnicity** |  |  |  |  |
| Turkish ethnicity | ref |  |  |  |
| Non-Turkish ethnicity | **0.80** | 0.33-1.96 | **-0.49** | 0.624 |
| **Cannabis use** | **5.66** | 1.93-16.54 | **3.16** | **0.002** |
| **Adversity** | **1.54** | 0.58-4.09 | **0.87** | 0.383 |
| **Trauma** | **0.96** | 0.42-2.19 | **-0.11** | 0.915 |
| **Family history of mental disorder** |  |  |  |  |
| None | ref |  |  |  |
| Unknown/other disorder | **1.59** | 0.32-7.82 | **0.57** | 0.567 |
| Common mental disorder | **3.39** | 1.37-8.35 | **2.65** | **0.008** |
| Severe mental disorder | **2.69** | 0.52-13.99 | **1.17** | 0.241 |

**PE**: Psychotic Experiences; **PD**: Psychotic Disorders; **OR**: Odds Ratio; **CI**: Confidence Interval

*No results because of too few data
