# Supplementary material for: The impact of affective and negative symptoms on the development of psychosis in a six-year follow-up of a community-based population
Source: Soc Psychiatry Psychiatr Epidemiol. 2024 Nov 7;60(6):1357–66. doi: 10.1007/s00127-024-02785-0 (PMC12162375; doi:10.1007/s00127-024-02785-0)
Supplement: Supplementary file 4 — Supplementary file4 (DOCX 27 KB) [file 127_2024_2785_MOESM4_ESM.docx]

**Supplement Table 4:** Results of the multinomial logistic regression analysis of the association between baseline negative symptoms and clinical characteristics with incident PE and PD at follow-up

|  | **Incident Subclinical PE** | | | | **Incident Clinical PE** | | | | **Incident PD** | | | |
| --- | --- | --- | --- | --- | --- | --- | --- | --- | --- | --- | --- | --- |
|  | **RR** | 95% CI | **z** | *p* | **RR** | 95% CI | **z** | *p* | **RR** | 95% CI | **z** | *p* |
| **Categories at T1** |  |  |  |  |  |  |  |  |  |  |  |  |
| No PE nor negative symptoms | **ref** |  |  |  | **ref** |  |  |  | **ref** |  |  |  |
| Negative symptoms | ***** | * | ***** | * | **6.90** | 2.73-17.48 | **4.08** | **0.001** | **7.81** | 0.80-76.77 | **1.76** | 0.078 |
| **Gender** |  |  |  |  |  |  |  |  |  |  |  |  |
| Male | **ref** | - |  |  | **ref** |  |  |  | **ref** |  |  |  |
| Female | **0.94** | 0.63-1.38 | **-0.34** | 0.736 | **1.51** | 0.93-2.44 | **1.66** | 0.097 | **0.57** | 0.15-2.14 | **-0.83** | 0.406 |
| **Age** |  |  |  |  |  |  |  |  |  |  |  |  |
| 15-30 | **ref** | - |  |  | **ref** |  |  |  | **ref** |  |  |  |
| 31-45 | **0.61** | 0.39-0.96 | **-2.14** | **0.032** | **0.85** | 0.50-1.46 | **-0.59** | 0.553 | **0.29** | 0.06-1.50 | **-1.48** | 0.140 |
| 46-65 | **0.53** | 0.33-0.85 | **-2.66** | **0.008** | **0.58** | 0.32-1.03 | **-1.85** | 0.064 | **0.28** | 0.05-1.45 | **-1.52** | 0.129 |
| **Ethnicity** |  |  |  |  |  |  |  |  |  |  |  |  |
| Turkish ethnicity | **ref** |  |  |  | **ref** |  |  |  | **ref** |  |  |  |
| Non-Turkish ethnicity | **0.76** | 0.49-1.19 | **-1.19** | 0.234 | **0.77** | 0.45-1.30 | **-0.99** | 0.323 | **0.69** | 0.14-3.33 | **-0.47** | 0.639 |
| **Cannabis use** | **3.73** | 1.37-10.13 | **2.58** | **0.010** | **2.72** | 0.72-10.32 | **1.47** | 0.141 | **3.48** | 0.33-36.07 | **1.04** | 0.298 |
| **Adversity** | **1.44** | 0.86-2.42 | **1.38** | 0.169 | **1.40** | 0.77-2.55 | **1.09** | 0.276 | **0.81** | 0.10-6.65 | **-0.20** | 0.844 |
| **Trauma** | **1.39** | 0.94-1.07 | **1.64** | 0.102 | **1.48** | 0.93-2.37 | **1.66** | 0.098 | **0.95** | 0.23-3.85 | **-0.08** | 0.939 |
| **Family history of mental disorder** |  |  |  |  |  |  |  |  |  |  |  |  |
| None | **ref** |  |  |  | **ref** |  |  |  | **ref** |  |  |  |
| Present | **0.88** | 0.53-1.47 | **-0.47** | 0.636 | **1.11** | 0.63-1.94 | **0.35** | 0.727 | **2.24** | 0.23-3.85 | **1.13** | 0.259 |

**PE**: Psychotic Experiences; **PD**: Psychotic Disorders; **RR**: Relative Ratio; **CI**: Confidence Interval

*No results because of too few data
